# Non-Invasive Detection of Prostate Cancer with Novel Time-Dependent Diffusion MRI and AI-Enhanced Quantitative Radiological Interpretation: PROS-TD-AI

**DOI:** 10.3390/jimaging12010053

**Published:** 2026-01-22

**Authors:** Baltasar Ramos, Cristian Garrido, Paulette Narváez, Santiago Gelerstein Claro, Haotian Li, Rafael Salvador, Constanza Vásquez-Venegas, Iván Gallegos, Víctor Castañeda, Cristian Acevedo, Gonzalo Cárdenas, Camilo G. Sotomayor

**Affiliations:** 1 Radiology Department, Clinical Hospital of the University of Chile, University of Chile, Independencia 8380453, Chile; baltasarramos@ug.uchile.cl (B.R.); cgarrido@hcuch.cl (C.G.); gcardenas@hcuch.cl (G.C.); 2 Urology Department, Clínica Dávila, Santiago 8431657, Chile; kikinarvaez@gmail.com; 3 School of Medicine, Faculty of Medicine, University of Chile, Santiago 8380453, Chile; santiago.gelerstein@ug.uchile.cl; 4 Key Laboratory for Biomedical Engineering of the Ministry of Education, College of Biomedical Engineering & Instrument Science, Zhejiang University, Yuquan Campus, 38 Zheda Road, Hangzhou 310027, China; haotianli@zju.edu.cn; 5 Radiology Department, Hospital Clinic, Universitat de Barcelona, 170, 08036 Barcelona, Spain; rsalvadoriz@gmail.com; 6 Laboratory for Scientific Image Analysis SCIAN-Lab, Integrative Biology Program, Institute of Biomedical Sciences ICBM, Faculty of Medicine, University of Chile, Santiago 8380453, Chile; covasquezv@inf.udec.cl; 7 Pathology Department, Clinical Hospital of the University of Chile, University of Chile, Independencia 8380453, Chile; igallegosmendez@gmail.com; 8 Medical Technology Department, Faculty of Medicine, University of Chile, Santiago 8380453, Chile; vcastane@uchile.cl; 9 Urology Department, Clinical Hospital of the University of Chile, University of Chile, Independencia 8380453, Chile; cacevedo@hcuch.cl; 10 Anatomy and Developmental Biology Program, Institute of Biomedical Sciences, Faculty of Medicine, University of Chile, Santiago 8380453, Chile; 11 Faculty of Medicine, San Sebastián University, Campus Los Leones, Santiago 7510157, Chile

**Keywords:** artificial intelligence, clinically significant prostate cancer, deep learning, Gleason score, magnetic resonance imaging, prostate biopsy, PI-RADS v2.1

## Abstract

Prostate cancer (PCa) is the most common malignancy in men worldwide. Multiparametric MRI (mpMRI) improves the detection of clinically significant PCa (csPCa); however, it remains limited by false-positive findings and inter-observer variability. Time-dependent diffusion (TDD) MRI provides microstructural information that may enhance csPCa characterization beyond standard mpMRI. This prospective observational diagnostic accuracy study protocol describes the evaluation of PROS-TD-AI, an in-house developed AI workflow integrating TDD-derived metrics for zone-aware csPCa risk prediction. PROS-TD-AI will be compared with PI-RADS v2.1 in routine clinical imaging using MRI-targeted prostate biopsy as the reference standard.

## 1. Introduction

### 1.1. Prostate Cancer Epidemiology

Prostate cancer (PCa) is a major global health challenge. The 2022–2027 National Cancer Plan’s official communication, based on GLOBOCAN data, reported that lung, prostate, and colorectal cancers are the three most common malignancies among men worldwide [1]. PCa is the most frequently diagnosed cancer in men in 118 of 185 countries. Cancer incidence varies according to socioeconomic development. Countries undergoing economic transition face an increased risk of certain cancers, particularly breast cancer in women and PCa in men. In Latin America, the overall cancer mortality rate is projected to increase by more than 100% in the coming years [1].

### 1.2. Overall Prostate Cancer Diagnostic Workflow

Risk-adaptive algorithms first combine prostate-specific antigen (PSA), digital rectal exam (DRE), prior biopsy, family history, and routine pre-biopsy multiparametric MRI (mpMRI). mpMRI classifies lesions with a Prostate Imaging–Reporting and Data System (PI-RADS) score from 1 to 5 to estimate the likelihood of clinically significant prostate cancer (csPCa). If the MRI is negative (PI-RADS 1–2) and clinical suspicion is low (e.g., PSA density < 0.20 ng/mL/cc; for PI-RADS 3, <0.10), biopsy will be deferred with PSA follow-up; otherwise, targeted and perilesional biopsy should be performed for higher scores (PI-RADS ≥ 4), in accordance with EAU–EANM–ESTRO–ESUR–ISUP–SIOG guidelines [2]. Prostate cancer aggressiveness is graded using the Gleason score (GS) or International Society of Urological Pathology (ISUP) grade groups. A score of ≥7 GS or ISUP ≥ 2 indicates highly cellular, clinically significant disease, which is more likely to progress and therefore requires active treatment [3].

### 1.3. Relevance of Non-Invasive Evaluation by mpMRI

Multiparametric MRI has reshaped prostate cancer care by detecting clinically significant disease while reducing unnecessary biopsies and overtreatment of clinically insignificant lesions [4,5,6]. Standard mpMRI combines T2-weighted (T2W), contrast-enhanced, and diffusion-weighted imaging, which are scored on the PI-RADS scale to estimate csPCa likelihood and guide biopsy decisions [7,8]. The updated PI-RADS v2.1 further improved tumor detection within the transition zone by 7–18 percentage points [9].

### 1.4. Diagnostic Limitations and Inter-Observer Variability in mpMRI

Despite these advances, mpMRI remains vulnerable to both false-negative and false-positive results. The inter-observer agreement for PI-RADS is moderate to substantial, with reported κ values of 0.62–0.70 for v2.0 and 0.70–0.72 for v2.1 [10,11]. Moreover, diagnostic accuracy varies widely across studies applying PI-RADS v2.1, with a reported sensitivity of 72–96.3%, specificity 62–93.5%, positive predictive value 53–78%, and negative predictive value 64–94.3% [12,13,14,15]. This variability hampers reliable interpretation for clinical decision-making. Further development of MRI techniques is needed to improve diagnostic performance, enabling accurate differentiation between benign diseases, non-clinically significant prostate cancer (non-csPCa), and csPCa, thereby reducing unnecessary biopsies and supporting cost-effective, imaging-based risk prediction.

### 1.5. Time-Dependent Diffusion MRI

Time-dependent diffusion (TDD) MRI is a relatively new sequence that probes water molecule diffusivity over short diffusion times, potentially revealing the tissue microstructure beyond single-cell resolution. Early animal studies demonstrated sensitivity to microscopic tumor features [16,17,18,19]; however, clinical adoption was limited by the need for very high gradient strengths [20,21].

Recent pilot studies on head and neck, breast, and prostate cancers demonstrated technical feasibility even with limited diffusion time ranges [22,23,24,25]. Using high-performance gradients (≤80 mT/m), Xu et al. advanced biophysical modeling to extract quantitative microstructural maps from TDD data; however, this approach was not yet feasible for routine diagnostics [23,26]. A breakthrough was achieved when Wu et al. showed that standard clinical MRI systems could acquire TDD images that distinguished csPCa from non-csPCa, exploiting the stronger diffusion-time dependence observed in highly cellular, high-grade tumors [27]. This line of research advances the concept of virtual prostate pathology using non-invasive imaging methods.

### 1.6. Further Possibilities with the TDD Sequence

Further validation of TDD microstructural parameters for PCa imaging must follow a staged evidence framework that confirms the following: (1) technical adequacy for better lesion detection; (2) diagnostic accuracy; (3) influence on physicians’ clinical reasoning; and (4) impact on patient management, culminating in outcome assessment within Fryback and Thornbury’s hierarchical model [28,29]. Independent real-world studies should avoid biases, such as the enriched prostatectomy sample used by Wu et al. [27], which may overestimate test performance. Finally, TDD invites the development of quantitative MRI metrics, moving beyond simple parameter averaging, to provide objective, low-variability tissue characterization that can inform clinical guidelines.

### 1.7. Deep Learning-Based Interpretation

Accurate lesion localization is essential because the peripheral and transition zones of the prostate differ markedly in architecture and cancer prevalence [30]. Advances in deep learning (DL) have enabled automated zone segmentation on both mpMRI and transrectal ultrasound, making zone-specific assessment feasible in routine practice [31,32].

Therefore, future work should evaluate zone-specific diagnostic performance of TDD-derived microstructural parameters and test machine learning algorithms for imaging-based PCa risk estimation, particularly given that reproducibility remains hampered by high inter-observer variability [33]. Within the standard diagnostic workflow, novel imaging techniques aim to reduce unnecessary biopsies by offering accurate and non-invasive detection of csPCa [34].

Accordingly, this study will prospectively assess the diagnostic contribution of TDD-derived imaging metrics integrated into an AI-based interpretation framework by comparing their performance in predicting csPCa against PI-RADS v2.1-driven mpMRI in real-world clinical practice. In line with this, the primary objective is to evaluate whether the AI-enhanced TDD-MRI pipeline (PROS-TD-AI) improves the classification of clinically significant prostate cancer (csPCa; ISUP grade group ≥ 2) within the PI-RADS 3–4 decision zone compared with standard PI-RADS v2.1 (with the primary endpoint being the paired specificity improvement for csPCa classification). The secondary objectives are as follows: (1) to develop and evaluate an automated prostate segmentation model (reporting the Dice similarity coefficient); (2) estimate TDD microstructural parameters and perform lesion/tissue classification (reporting AUC, accuracy, sensitivity, and specificity with 95% CIs); and (3) assess the integrated end-to-end PROS-TD-AI pipeline combining imaging and clinical features, including robustness and failure modes.

## 2. Materials and Methods

### 2.1. Study Design

This is a prospective, observational, and analytical study, incorporating an exploratory component aimed at developing and validating a novel MRI protocol and an AI-based interpretation framework named PROS-TD-AI DL for prostate zonal segmentation and lesion classification. This study will be conducted at the Clinical Hospital of the University of Chile (HCUCH) and the Faculty of Medicine of the University of Chile. This protocol follows a paired (within-subject) diagnostic–accuracy comparison, as each participant contributes both the standard PI-RADS assessment and PROS-TD-AI output, with biopsy used as the reference standard when performed. The protocol is approved by the Institutional Review Board (or Ethics Committee) of the University of Chile Clinical Hospital (protocol code 1339/23, approved on 9 May 2023). No commercial entity participated in the design of this study, nor will any be involved in its execution or analysis. Reporting will follow the STARD-AI guideline for diagnostic accuracy studies involving artificial intelligence; a completed STARD-AI checklist with page/section references is provided in Supplementary Table S1 [35].

### 2.2. Study Population

**Figure 1:**
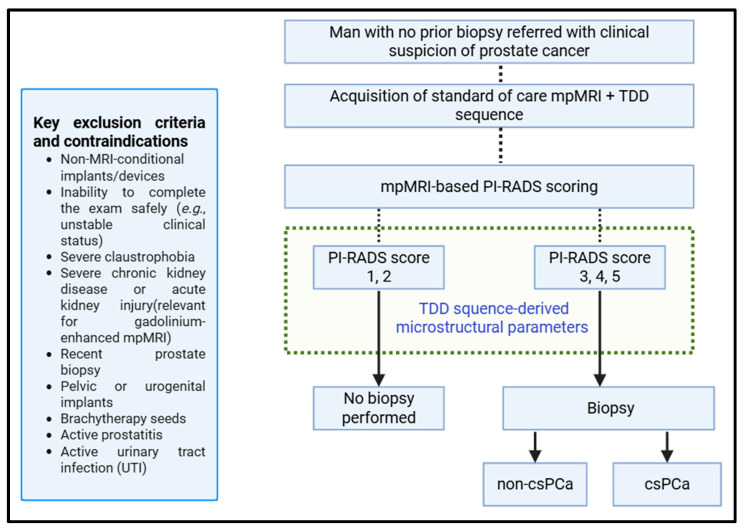
Study flow and imaging protocols. Key exclusion criteria and contraindications are listed on the left. TDD-derived microstructural parameters will be extracted from all patients undergoing mpMRI. mpMRI, multiparametric MRI; TDD, time-dependent diffusion; PI-RADS, Prostate Imaging–Reporting and Data System; csPCa, clinically significant prostate cancer; non-csPCa, non-clinically significant prostate cancer.

Consecutive patients with clinical suspicion of PCa—defined as an elevated prostate-specific antigen (PSA) level > 4 ng/mL, an abnormal digital rectal examination, or both, without prior prostate biopsy—will be recruited from outpatient clinics. Eligibility criteria, key contraindications, and the imaging workflow, including acquisition of the TDD sequence, are summarized in Figure 1. All participants will provide written informed consent prior to enrollment.

According to the local standard-of-care diagnostic pathway, men with clinical suspicion of PCa undergo mpMRI (including T2-weighted (T2W), diffusion-weighted (DWI), and dynamic contrast-enhanced (DCE) sequences). The dominant and any additional PI-RADS ≥ 3 lesions are scored by a senior radiologist with at least 10 years of experience using the PI-RADS v2.1 system:

- PI-RADS 1: Very low (clinically significant cancer highly unlikely).
- PI-RADS 2: Low (clinically significant cancer unlikely).
- PI-RADS 3: Intermediate (equivocal).
- PI-RADS 4: High (clinically significant cancer likely).
- PI-RADS 5: Very high (clinically significant cancer highly likely).

Patients with PI-RADS 1–2 findings are not offered biopsy, while those with PI-RADS 3–5 lesions undergo prostate biopsy as per standard of care, following the Ginsburg protocol [36]. Biopsy results are interpreted according to the EAU–EANM–ESTRO–ESUR–ISUP–SIOG guidelines [2] and serve as the diagnostic reference standard for this study.

For the purpose of this study, all enrolled patients will undergo an additional 4.5-minute TDD sequence during the same visit as the standard mpMRI. All image data will be exported to a dedicated database. Only standard-of-care mpMRI sequences will be stored in the institutional Picture Archiving and Communication Systems (PACS) for clinical use. We will report baseline clinical and imaging characteristics (e.g., age, PSA, PSA density, prostate volume, prior MRI findings, and scanner/protocol variables) for the full cohort and, where applicable, by analysis split (training/validation/test) and by PI-RADS category.

### 2.3. Input-Data Eligibility and Non-Evaluable Examinations

For a case to be evaluable for the primary analysis, mpMRI must include diagnostically adequate T2W, DWI (including high b-value), and DCE, plus the additional TDD sequence acquired per protocol with complete prostate coverage. An examination will be classified as non-evaluable for the primary analysis if any of the following prespecified conditions occur: (one) the magnetic resonance imaging examination is incomplete or prematurely terminated; (two) time-dependent diffusion acquisition fails or is not acquired according to the prespecified protocol; (three) severe motion, susceptibility, or other artifacts prevent reliable region of interest definition or parameter extraction for the index lesion; (four) prostate gland or lesion segmentation fails despite prespecified fallback procedures; (five) biopsy verification for the PI-RADS category 3–4 primary analysis cohort is not obtained within the prespecified verification window due to patient refusal, contraindication, loss to follow up, or clinical decision changes. Non-evaluable cases will be reported with reasons and accounted for in the participant flow and sensitivity analyses.

### 2.4. Prostate Magnetic Resonance Imaging Data Acquisition and Fitting for Determination of Microstructural Parameters

Multiparametric MRI will be performed on a 3.0 T scanner with the participant in the supine position using a pelvic phased-array coil with or without an endorectal coil. Standard mpMRI sequences (T2W, DWI, and DCE) will be acquired following consensus guidelines [37].

An additional TDD sequence will be acquired following the protocol proposed by Wu et al. [27]. This protocol uses oscillating gradient spin-echo frequencies of 33 Hz (effective diffusion time = 7.5 ms, two cycles, b = 300 and 600 s/mm^2^) and 17 Hz (effective diffusion time = 15 ms, one cycle, b = 400, 800, and 1200 s/mm^2^) [27], and pulsed gradient spin-echo sequences at diffusion duration and separation of 10 and 30 msec, respectively (effective diffusion time = 26.7 ms, b = 400, 800, and 1200 s/mm^2^), with a maximum gradient of 45 mT/m and maximum slew rate of 200 mT/m/msec [27].

Diffusion data will be analyzed with a two-compartment model with impermeable spheres [26], fitted by non-linear least squares optimization in Matlab (Version R2025b) (MathWorks). To avoid local minima, the fitting will be repeated 20 times with randomized initialization. The signal model is given by the following:

(1) $$S=v_{in}\cdot S_{in}\cdot \left(1-v_{in}\right)\cdot e^{-b\cdot D_{ex}}$$

where $v_{in}$ is the intracellular volume fraction; $S_{in}$ is the intracellular signal (as defined by Jiang et al. [26] for trapezoidal OGSE encoding [23]); ***b*** is the diffusion-weighting factor; and ***D_ex_*** is the extracellular diffusivity [19,26]. The Matlab routine provided by Jiang et al. [38] will be used.

### 2.5. Artificial Intelligence-Based Automatic Delineation of the Prostate Gland Zones

This study aims to develop and validate an AI-based framework for the automatic delineation of prostate zones and suspicious lesions, enabling accurate three-dimensional quantification of microstructural parameters for the prediction of csPCa.

#### 2.5.1. Training Dataset and Human-in-the-Loop Strategy

DL-based segmentation requires annotated data. We will begin with the publicly available PI-CAI dataset [39] and its annotations [40]. The relevance of this dataset is that it comprises mpMRI examinations collected for addressing challenges around prostate lesion detection and classification. It provides T2W, DWI, and apparent diffusion coefficient (ADC) images acquired on Siemens scanners, along with lesion annotations and clinical significance labels (biopsy-confirmed Gleason Grade Group) for a subset of patients. For local patient data, a Human-in-the-Loop strategy will be implemented: batches of automatic segmentations from a “no new U-Net” nnU-Net trained on the public dataset will be iteratively corrected by expert radiologists [41,42]. Corrected masks are added to the training set, and the model is retrained until convergence, defined by radiologist-established criteria. Model performance will be evaluated on an unseen test set using the Dice coefficient or more sophisticated Dice variants measurements.

#### 2.5.2. Segmentation Models

Automatic segmentation will primarily rely on nnU-Net [42], a self-configuring deep learning framework that automatically adapts pre-processing, network architecture, training schedules, and post-processing to the characteristics of each dataset. This approach is based on the U-Net architecture, employing a contracting path to capture contextual information and an expanding path to delineate regions of interest with high precision. For additional flexibility and integration of custom architectures, we will also leverage the Medical Open Network for Artificial Intelligence (MONAI) framework [43]. MONAI (v1.5.1) is a PyTorch-based open-source framework (PyTorch v2.9.1) that provides medical imaging-specific transforms, model architectures, and utilities to streamline the development and deployment of clinical AI models. Together, nnU-Net and MONAI establish a robust, state-of-the-art segmentation pipeline that is reproducible and adaptable to diverse imaging datasets. The primary segmentation metric will be the Dice coefficient. All models will be evaluated on the same prespecified, patient-level held-out test set—locked prior to training and never used for model development—hyperparameter tuning, or early stopping to ensure fair, like-for-like comparisons.

In addition to these baselines, we will consider foundation models such as the Medical Segment Anything Model (MedSAM), which leverages large-scale pre-training for cross-domain adaptability and can be adapted for automatic use via lightweight fine-tuning or prompt generation [44]. MedSAM is trained on over 1.5 million annotated medical images and significantly improves segmentation performance across a wide variety of anatomical structures and imaging modalities. We will also evaluate ProGNet [45], a U-Net derivative optimized for automatic prostate segmentation. ProGNet has been retrospectively validated on 905 patients and prospectively tested, achieving mean Dice coefficients of 0.92 and 0.93, respectively, while reducing segmentation time from >10 min manually to ~35 s per case. Its code is publicly available for reproducibility and external validation [45,46].

#### 2.5.3. Integration with Microstructural Analysis

Automatic segmentations will define all regions of interest (ROIs) for TDD-derived microstructural parameter extraction. Combined with biopsy results, these metrics will be used to train the PROS-TD-AI DL model for lesion-level risk prediction, aiming to improve reproducibility, reduce inter-observer variability, and support clinical decision-making.

#### 2.5.4. Model Governance

- Versioning: All AI components (segmentation, parameter estimation, and classification) will be version-controlled (code, pre-processing, and trained weights). The final selected model will be locked (frozen parameters) prior to final evaluation, and any subsequent modifications will be released as a new version and evaluated separately.
- Bias Mitigation: To mitigate bias and leakage, data splitting is performed at the patient level only, with stratification by csPCa status; all pre-processing, feature selection, calibration, and threshold tuning will occur within training folds. We will report performance stratified by clinically relevant subgroups (e.g., peripheral vs. transition zone; PI-RADS category) to assess heterogeneity.
- Explainability: The system will output clinician-facing results, including ROI overlays and lesion-level probability scores. For model transparency, we will report feature importance/attribution (e.g., permutation importance or SHAP for tree-based models) and calibration summaries, while emphasizing that explainability outputs are supportive and not used for ground-truth determination.
- Monitoring: We will prospectively log non-evaluable cases and failure modes (e.g., missing sequences, motion/artifacts, and segmentation/fit failure) and report their frequency and impact on performance. During this observational study, PROS-TD-AI outputs will not influence clinical management.

### 2.6. Radiological Imaging Analysis

All standard mpMRI examinations (excluding the TDD sequence) will be interpreted according to PI-RADS v2.1 by radiologists through PACS. After signing the report, the radiologist will segment the index lesion and any additional PI-RADS 3-4 lesions within one month, blinded to biopsy results. These segmented regions will define the ROIs for extracting microstructural parameters from co-registered TDD images. TDD-derived maps will not be displayed during lesion contouring.

Microstructural parameters—including intracellular volume fraction (v***_in_***), cell diameter (***d***), extracellular diffusivity (***D_ex_***), and cellularity index (v***_in_/d***)—will be estimated using Microstructure Estimation Transformer with Sparse Coding (METSC) [47], a transformer-based architecture designed for multi-shell diffusion MRI. METSC addresses limitations of conventional non-linear optimization in biophysical multi-compartment models by learning latent representations directly from q-space samples. This approach reduces the number of diffusion encodings required, demonstrating reduced scan times while preserving or improving fitting accuracy.

TDD-derived microstructural metrics obtained using METSC will be evaluated for their ability to classify lesion categories and will contribute to the training and validation of the PROS-TD-AI framework.

### 2.7. Histopathologic Analysis

The interval between MRI examination and prostate biopsy will be limited to 30 days. All biopsy specimens will be interpreted by senior pathologists with over 10 years of experience, following the International Society of Urological Pathology (ISUP) grading standards [48]. The GSs of all tumor foci will be recorded and the findings grouped into five categories according to the ISUP grade groups (GGs): GS 3+3 (GG 1), GS 3+4 (GG 2), GS 4+3 (GG 3), GS 4+4 (GG 4), and GS over 4+4 (GG 5). For analysis purposes, benign lesions and PCa GG 1 will be classified as non-clinically significant prostate cancer (non-csPCa), while lesions within GGs 2–5 will be classified as csPCa. To link imaging findings to the reference standard, MRI-visible targets (index lesion and any additional PI-RADS ≥ 3 lesions) will be contoured and assigned standardized lesion identifiers and location labels. During MRI-guided biopsy, each targeted core will be recorded with the corresponding lesion identifier, enabling a core-level linkage between MRI targets and histopathology. When multiple targeted cores are obtained for a lesion (and when systematic cores are also available), the lesion-level classification will be determined using the highest ISUP grade group among the cores assigned to that MRI target, with predefined rules to adjudicate discordant or multifocal findings. Among csPCa cases, we will report the distribution of ISUP grade groups (1–5) and summarize lesion-level grade where applicable. Among non-csPCa participants, we will report alternative diagnoses, including benign histology and ISUP 1 (non-clinically significant cancer), and any other relevant findings (e.g., prostatitis/benign prostatic hyperplasia) when available.

### 2.8. PROS-TD-AI Output

PROS-TD-AI will provide a clinician-facing csPCa risk score (0–1) at the patient level and, when applicable, at the lesion level for PI-RADS 3–4 assessments. It will display prostate/lesion segmentation overlays and lesion probability maps co-registered with standard MRI sequences. Using prespecified, locked decision cutoffs (upgrade and downgrade cutoffs), it will recommend whether to maintain PI-RADS 3–4 or reclassify between PI-RADS 3 and 4 (3 → 4 or 4 → 3); however, these recommendations will not alter clinical management during this observational study.

### 2.9. Statistical Analysis

Microstructural parameters will be measured within manually delineated lesion ROIs, excluding boundary voxels to minimize partial-volume effects. In addition to mean values, we will compute a small prespecified set of alternative ROI summary statistics (e.g., median, selected percentiles, and dispersion metrics), with any feature-set selection performed strictly within the training data to avoid optimistic bias.

Post hoc comparisons among tissue categories (benign tissue and ISUP grade groups) will be performed using one-way ANOVA (or the Kruskal–Wallis test if assumptions are violated), followed by multiplicity-adjusted pairwise testing. Analyses will be conducted using RStudio (Version IDE 2026.01.0) and IBM SPSS Statistics (Version 31.0.1.0) software.

Diagnostic performance will be evaluated in the biopsy-verified PI-RADS 3–4 cohort (csPCa defined as ISUP ≥ 2). We will train and compare LDA, SVM, Random Forest, and XGBoost, selected to represent linear, margin-based, and ensemble tree-based approaches with different bias–variance trade-offs, using patient-level, stratified fivefold cross-validation with all pre-processing and hyperparameter tuning performed within training folds only. Model selection will be prespecified and specificity-driven. Among candidate classifiers, we will prioritize the approach that maximizes specificity for non-csPCa within the PI-RADS 3–4 cohort, while maintaining sensitivity above a prespecified minimum for csPCa. The upgrade and downgrade thresholds will be determined within training folds only using this prespecified criterion, then locked and applied unchanged to the held-out/prospective evaluation cohort. Performance will be reported with 95% confidence intervals.

PROS-TD-AI will output two continuous risk scores: an upgrade risk (0–1) for PI-RADS 3 cases (likelihood that the case should be managed as PI-RADS 4/csPCa-likely), and a downgrade risk (0–1) for PI-RADS 4 cases (likelihood that the case should be managed as PI-RADS 3/non-csPCa-likely). Prespecified, locked thresholds (the prespecified upgrade cutoff and the prespecified downgrade cutoff) will map these scores to a binary recommendation within the PI-RADS 3–4 decision zone (final recommendation: “3” vs. “4”), corresponding to non-csPCa-likely versus csPCa-likely classification in this subgroup.

The primary endpoint is the paired improvement in specificity (non-csPCa correctly recommended as “3”) of PROS-TD-AI-assisted recommendations versus standard PI-RADS (4 vs. 3), tested using McNemar’s test [49], in the non-csPCa subgroup (two-sided alpha = 0.01). The secondary endpoints include paired sensitivity in csPCa (McNemar), upgrade/downgrade reclassification rates stratified by pathology, and continuous-score discrimination (ROC AUC) and calibration (*p* < 0.05 for secondary analyses). We will present 2 × 2 cross-tabulations (index test vs. reference standard) for PI-RADS and PROS-TD-AI recommendations in the biopsy-verified PI-RADS 3–4 cohort, including counts of TP, FP, TN, and FN. Baseline demographic, clinical, and technical characteristics will be summarized for each analysis split (training/validation/test and the prospective evaluation cohort) to assess representativeness and potential distribution shift, including age, PSA/PSA density, prostate volume, PI-RADS category, and key acquisition variables (scanner/vendor and sequence quality markers). Because participants are consecutively enrolled within the routine intended-use pathway (biopsy-verified PI-RADS 3–4), we expect the csPCa prevalence to reflect this clinical setting. We will report the observed csPCa prevalence and discuss any material deviation as potential spectrum effects affecting generalizability.

### 2.10. Sample Size Calculation

The primary endpoint is the within-participant difference in specificity for detecting clinically significant prostate cancer (csPCa; ISUP ≥ 2) within the PI-RADS 3–4 decision zone, comparing standard PI-RADS v2.1 assessment (test-positive defined as PI-RADS 4; test-negative as PI-RADS 3) versus the PROS-TD-AI-assisted recommendation (positive = “recommend 4”; negative = “recommend 3”). All eligible PI-RADS 3–4 examinations will undergo MRI-targeted biopsy (±systematic biopsy per local protocol) within a prespecified time window, providing complete histopathological verification for the primary analysis. We powered this study to detect an absolute specificity improvement of 0.10 (two-sided alpha = 0.01; 80% power) using a paired-proportions (McNemar) approach applied to the non-csPCa subgroup. Let p10 denote the proportion of non-csPCa participants classified as positive by PI-RADS (4) but negative by PROS-TD-AI (3) (false-positive→true-negative), and p01 denote the reverse discordance. Under conservative, prespecified discordant-pair assumptions (p10 = 0.15; p01 = 0.05; net delta = p10 − p01 = 0.10; total discordance = p10 + p01 = 0.20), the required number of non-csPCa participants is computed as follows: n_non-csPCa = ((z_(1−alpha/2) + z_(1−beta))^2^ × (p01 + p10))/(p10 − p01)^2^. Using z_(1−alpha/2) = 2.576 and z_(1−beta) = 0.842 gives n_non-csPCa ≈ ((2.576 + 0.842)^2^ × 0.20)/0.10^2^ ≈ 234 [49]. To convert this to the total sample size, we used the PROMIS trial as an external prevalence benchmark; PROMIS reported 220/576 = 38% (95% CI 34–42) clinically significant cancer on template mapping biopsy [6]. For conservative planning, we assumed 38% (the proportion of ISUP csPCa, thus 62% non-csPCa), yielding N_total = 234/0.62 ≈ 378 PI-RADS 3–4 participants. We will inflate this by 10–15% for exclusions and non-evaluable examinations, resulting in a planned enrollment of approximately 420–445 participants (target ~450). This target also satisfies empiric minimum sample sizes proposed for objective testing of AI binary classification performance metrics (including ROC AUC), where an empirical minimum sufficient test sample size of approximately 400 studies has been reported [50].

### 2.11. Cross Validation

All model development will use patient-level, stratified, grouped cross-validation to prevent data leakage: all images, sequences, lesions, and derived patches from a given patient will be assigned to a single fold only (no lesion/slice-level splitting). Folds will be stratified by csPCa status (ISUP ≥ 2) (and, where feasible, by PI-RADS category) to preserve class balance. Any data-dependent pre-processing and model selection steps (e.g., normalization/scaling, imputation, feature selection, threshold tuning, and calibration) will be performed exclusively within the training folds and then applied unchanged to the corresponding validation folds. The prospective cohort will be reserved for final evaluation only and will not be used for training, hyperparameter tuning, or threshold selection. The final model will be locked prior to prospective enrollment.

## 3. Expected Results

### 3.1. Deep Learning Models

We anticipate the development of two complementary deep learning models for automated prostate analysis:

- Prostate Segmentation Model: Based on U-Net or ProGNet architectures, initially trained on the PI-CAI dataset [39] and subsequently fine-tuned using multiparametric MRI (mpMRI) data from the Clinical Hospital of the University of Chile (HCUCH). This model is expected to accurately delineate the prostate gland and serve as a pre-processing step for downstream microstructural analysis. For the segmentation task, a Dice similarity coefficient of ~0.92 is an a priori target informed by the prior literature; observed segmentation performance will be reported accordingly.
- Tissue Microstructure Estimation Model: Employing a transformer-based architecture inspired by sparse representation techniques; METSC [47], this model will estimate voxel-wise tissue microstructural parameters—including intracellular and extracellular volume fractions and diffusivities—from multi-shell diffusion MRI (dMRI) data. These microstructural parameters will then be used for tissue classification. For microstructure estimation and lesion classification, we hypothesize clinically meaningful discrimination and will report AUC, accuracy, sensitivity, specificity, and 95% confidence intervals in the final study report. Any numerical performance figures stated here are a priori targets informed by the prior literature rather than observed outcomes.

Together, these models will form a fully automated pipeline integrating anatomical and microstructural information to support clinical decision-making in PCa diagnostics.

### 3.2. Pipeline Integration

We propose an end-to-end deep learning pipeline that combines anatomical segmentation with microstructure-based tissue classification. The pipeline will be trained end-to-end using a deep neural network that combines imaging data with clinical features, thereby simultaneously optimizing segmentation and classification objectives. This joint learning approach enables shared feature representation, enhancing both anatomical delineation and microstructural characterization. By integrating diffusion-informed biomarkers with anatomical information, this framework is expected to improve diagnostic accuracy and provide clinically meaningful predictions.

## 4. Discussion

This protocol is designed to prospectively develop and evaluate novel imaging techniques and analytic approaches aimed at improving non-invasive detection of csPCa. By integrating time-dependent diffusion MRI (TDD-MRI) with AI-based quantitative analysis, this study seeks to enhance diagnostic specificity, reduce the reliance on invasive biopsy procedures, and provide accurate, low-risk, and more accessible risk stratification for men at intermediate clinical risk.

Demonstrating higher specificity and reliable performance of these novel imaging methods could have a significant impact on both research and clinical practice. Positive findings would provide a foundation for future randomized controlled trials and support evidence-based updates to current standard-of-care diagnostic workflows, potentially leading to more personalized and efficient patient management.

PROS-TD-AI may offer its greater incremental clinical value in diagnostic scenarios where mpMRI interpretation is most equivocal and PI-RADS exhibits lower specificity, particularly for PI-RADS 3–4 [51] lesions and transition-zone/central gland abnormalities, in which benign prostatic hyperplasia and stromal changes can closely mimic clinically significant disease [9]. In these settings, the addition of TDD-derived microstructural biomarkers and quantitative AI-based analysis may improve discrimination and reduce inter-reader variability, thereby refining risk stratification around biopsy decision thresholds and potentially decreasing unnecessary biopsies without compromising sensitivity for csPCa diagnosis.

In addition to advances in non-invasive cancer detection techniques, there is a growing trend toward biparametric MRI (bpMRI) over mpMRI to reduce scan duration, lower costs, and avoid gadolinium without losing accuracy. The multicenter PRIME trial showed non-inferiority of bpMRI for detecting csPCa compared to mpMRI (29.2% vs. 29.6%, respectively), with 99% adequate quality scans, aligning with wider evidence that contrast-free protocols can maintain accuracy while improving efficiency [52,53,54]. Nevertheless, this study retains mpMRI (including DCE) as the guideline-concordant reference for model development and evaluation. Given that TDD-MRI does not require contrast, it could strengthen future bpMRI protocols by adding microstructural information; training AI on bpMRI + advanced diffusion is a promising future direction. Prospective head-to-head studies and external validation are still needed to confirm whether contrast-free pipelines can match or surpass mpMRI in routine clinical practice.

From an implementation standpoint, several barriers may influence the clinical adoption of this approach. The TDD sequence was developed and optimized on a specific 3.0 T scanner platform (Skyra, Siemens Healthcare), and its portability to other scanners, vendors, field strengths, or acquisition environments may require sequence harmonization, rigorous quality control, and external validation. In addition, TDD-derived microstructural estimates can vary with scanner hardware and vendor-specific implementation details, gradient performance, and acquisition settings, as well as with signal quality and motion sensitivity; these factors may affect quantitative feature stability and limit transportability of models trained under a single configuration. To mitigate these risks, the protocol prespecifies acquisition standardization, systematic quality control procedures, and detailed reporting of scanner characteristics and acquisition parameters to support interpretability, replication, and future multi-site evaluation. In addition, successful deployment will depend on adequate computational resources and seamless integration into clinical workflows (e.g., PACS/viewer overlays and reporting), as well as training for radiologists and technologists to interpret risk scores and probability maps and to apply PROS-TD-AI recommendations appropriately. Other limitations include the single-center setting, potential spectrum effects inherent to a biopsy-verified PI-RADS 3–4 cohort, and the need for external multi-vendor validation before broad clinical adoption. As an observational study, PROS-TD-AI outputs will not modify management; therefore, downstream clinical impact will require future interventional evaluation.

This project emphasizes the translational value of basic and preclinical research into clinically actionable applications. By focusing on clinically meaningful endpoints, it bridges the gap between advanced imaging research and real-world patient care, supporting evidence-driven decision-making in prostate cancer management.

## Data Availability

The original contributions presented in this study are included in the article and the Supplementary Materials. Further inquiries can be directed to the corresponding author.

## Supplementary Materials

The following supporting information can be downloaded at: https://www.mdpi.com/article/10.3390/jimaging12010053/s1, Supplementary Table S1: STARD-AI Checklist; Supplementary Material S1: PROSTDAI Study—Informed Consent Form.

## Abbreviations

The following abbreviations are used in this manuscript:

PCa	Prostate cancer
PSA	Prostate-specific antigen
DRE	Digital rectal exam
mpMRI	Multiparametric MRI
csPCa	Clinically significant prostate cancer
GS	Gleason score
ISUP	International Society of Urological Pathology
Non-csPCa	Clinical insignificant prostate cancer
TDD	Time-dependent diffusion
GG	Gleason grade
