# Supplementary material for: Non-Invasive Detection of Prostate Cancer with Novel Time-Dependent Diffusion MRI and AI-Enhanced Quantitative Radiological Interpretation: PROS-TD-AI
Source: J Imaging. 2026 Jan 22;12(1):53. doi: 10.3390/jimaging12010053 (PMC12843277; doi:10.3390/jimaging12010053)
Supplement: Supplementary file 1 [file jimaging-12-00053-s001.zip › jimaging-4049221-supplementary.pdf]

Supplementary Table S1: STARD-AI Checklist

For the official wording, consult: Nature Medicine (2025) “The STARD-AI reporting guideline for diagnostic accuracy studies using artificial intelligence [35]”.

ITEM	WHAT TO REPORT	WHERE IN MY PROTOCOL/MANUSCRIPT (PAGE/SECTION)	NOTES / ACTION
**1**	State in the title/abstract that this is an AI-based diagnostic accuracy study; include ≥1 accuracy metric.	Page 1-2/ section Abstract.	Completed
**2**	Provide a structured abstract covering design, methods, results, and conclusions (per STARD for Abstracts).	Page 2/section Abstract	Completed
**3**	Describe the clinical background and intended use of the AI test, including workflow placement and whether novel/established.	Pages 2-4/ section Introduction	Completed
**4**	Specify study objectives and hypotheses.	Page 4/ section Introduction	Completed
**5**	State whether data collection was planned before testing (prospective) or after (retrospective).	Page 4/ section Materials and Methods	Completed
**6**	Report ethics approval (or justify why not required).	Page 4/ section Materials and Methods	Completed
**7**	List inclusion/exclusion criteria and the order they are applied (participant- and data-level).	Pages 4-6/ section Materials and Methods	Completed
**8**	Explain how eligible participants were identified (e.g., symptoms, prior tests, registry inclusion).	Page 4-5/ section Materials and Methods	Completed
**9**	Report setting/location and relevant dates (where/when participants were identified).	Page 5/ section Materials and Methods	Location completed; dates are not applicable
**10**	State whether recruitment was consecutive, random, or convenience.	Page 4-5/ section Materials and Methods	Completed
**11**	Describe data source and whether routine, purpose-collected, or open-source.	Pages 4-6/ section Materials and Methods	Completed; routine collected
**12**	Describe who annotated the data (background/experience) and how/when annotations were done.	Pages 6-9 / section Materials and Methods	Completed
**13**	Report acquisition devices (manufacturer/model) and software used to build/run the AI test (with versions); clarify intended use.	Pages 6-8/ section Materials and Methods	Completed

**14**	Report acquisition protocols and preprocessing/quality control steps relevant to the index test.	Pages 6-9/ section Materials and Methods	Completed
**15A**	Describe the AI index test in enough detail to replicate.	Pages 6-11/ section Materials and Methods	Completed
**15B**	Describe model development/evaluation (training/validation/test/external), with sample sizes where applicable.	Pages 6-11/ section Materials and Methods	Completed
**15C**	Define and justify positivity thresholds/decision rules for the AI test; distinguish prespecified vs exploratory.	Pages 6-11/ section Materials and Methods	Completed
**15D**	Specify intended end-user and required expertise/training.	Page 9/ section Materials and Methods	Completed
**16A**	Describe the reference standard in enough detail to replicate.	Pages 8-9/ section Materials and Methods	Completed
**16B**	Justify choice of reference standard if alternatives exist.	Pages 8-9/ section Materials and Methods	Completed
**16C**	Define and justify reference-standard positivity thresholds/rules; prespecified vs exploratory.	Pages 8-9 / section Materials and Methods	Completed
**17A**	State whether index-test readers/developers had access to clinical info and/or reference-standard results.	Page 8/ section Materials and Methods	Completed
**17B**	State whether reference-standard assessors had access to clinical info and/or index-test results.	Pages 8-9/ section Materials and Methods	Completed
**18**	Describe statistical methods to estimate and/or compare diagnostic accuracy.	Pages 9-12/ section Materials and Methods	Completed
**19**	Explain how indeterminate results (index test or reference) were handled.	Page 6/ section Materials and Methods	Completed
**20**	Explain how missing data were handled (index test and/or reference).	Page 6/ section Materials and Methods	Completed
**21**	Describe analyses of variability/subgroups; prespecified vs exploratory.	Pages 9-10/ section Materials and Methods	Completed
**22**	State intended sample size and how it was determined.	Page 10/ section Materials and Methods	Completed
**23**	Describe error analysis and any bias/fairness assessments (if performed).	Pages 9-10/ section Materials and Methods	Completed

**24**	Provide a participant flow diagram.	Page 5/ section Materials and Methods	Completed
**25**	Report baseline demographic/clinical/technical characteristics for training/validation/test sets (if applicable).	Page 10/ section Materials and Methods	Completed
**26A**	Report distribution of disease severity among those with the target condition.	Page 9 /section Material and Methods	Completed
**26B**	Report alternative diagnoses among those without the target condition.	Page 9 /section Material and Methods	Completed
**27**	Report time interval and any interventions between index test and reference standard.	Page 8/ section Materials and Methods	Completed
**28**	State whether datasets reflect the target-condition distribution expected in the intended-use population.	Page 10/ section Materials and Methods	Completed
**29**	If externally evaluated, describe how the external dataset differs from train/val/test sets.	Not applicable	Not applicable
**30**	Provide cross-tabulation of index-test results versus reference standard (or distributions if continuous).	Page 10/ section Materials and Methods	Completed
**31**	Report accuracy estimates with precision (e.g., 95% CIs).	Page 11/section Expected Results	Completed
**32**	Report any adverse events from the index test and/or reference standard.	Not applicable	Not applicable
**33**	Discuss limitations: bias sources, uncertainty, and generalizability.	Pages 12-13 /section Discussion	Completed
**34**	Discuss implications for practice: intended use and clinical role of the AI test.	Page 12/ section Discussion	Completed
**35**	Describe AI-specific ethical considerations, including fairness/inequities and responsible use.	Pages 7-9, 13/ section Materials and Methods, informed consent	Completed
**36**	Provide registration number and registry name (if registered).	Not applicable	Not applicable
**37**	State where the full protocol can be accessed.	PAGE 13	Completed
**38**	Report funding/support and the funders' role.	PAGE 13	Completed
**39**	Declare commercial interests (if any).	Not applicable	Not applicable
**40A**	State availability of datasets/code and any reuse restrictions.	PAGE 13	Completed
**40B**	State whether outputs are stored/auditable and can be evaluated if needed.	Not applicable	Not applicable

Supplementary Material S1: PROSTDAI Study—Informed Consent Form

Project Title: *Non-Invasive Detection of Prostate Cancer with Novel Time-Dependent Diffusion MRI and AI-Enhanced Quantitative Radiological Interpretation (PROSTDAI)*

Principal Investigator: Dr. **[Name]** – *[Department]*, Clinical Hospital of the University of Chile (HCUCH)

Institution: Clinical Hospital of University of Chile / Faculty of Medicine, University of Chile

Contact Phone: [Phone Number] (24-hour contact if applicable)

Sponsor: *None (Investigator-initiated study)*

Invitation to Participate

You are invited to participate in a research study titled **"Non-Invasive Detection of Prostate Cancer with Novel Time-Dependent Diffusion MRI and AI-Enhanced Quantitative Radiological Interpretation (PROS-TD-AI)."** We are inviting you to join this study because you have clinical indicators that suggest a risk of prostate cancer (for example, an elevated prostate specific antigen (PSA) blood test or an abnormal prostate exam). **Before you decide whether to participate, please read this document carefully.** Feel free to ask any questions you may have about the study. Your participation is entirely voluntary.

Introduction and Background

This study involves a new **magnetic resonance imaging (MRI) technique** and an artificial intelligence (AI) software aimed at improving prostate cancer detection. Currently, men with suspected prostate cancer undergo a standard MRI of the prostate (called a *multiparametric MRI*) to look for suspicious areas. However, even with standard MRI, some significant cancers can be missed or mistaken for non-cancerous conditions.

The PROSTDAI study is evaluating an **experimental MRI sequence** (called *time-dependent diffusion MRI*) and an AI-based image analysis. This new MRI sequence takes additional detailed images of the prostate's microscopic structure. The AI software will analyze these images to assist in identifying prostate cancer. The knowledge gained from this research may help develop more accurate, computer-assisted MRI interpretations in the future, potentially improving diagnosis for future patients. Importantly, this is an **observational study**: the experimental MRI and AI analysis will **not** influence your

immediate medical care or treatment decisions. All your standard medical care will proceed as usual, regardless of study participation.

Study Objectives

The main objective of this study is **to determine whether adding the new time-dependent diffusion MRI sequence and AI analysis can improve the detection of clinically significant prostate cancer compared to the standard MRI alone.** We will assess how well the AI-enhanced MRI can identify cancerous tissue by comparing its findings with the results of the routine diagnostic procedures (such as standard MRI assessments and prostate biopsy results).

This research is **prospective and observational**, meaning we observe and collect data during your routine care without altering your treatment. Approximately **[450]** men will be enrolled in this study at the Clinical Hospital University of Chile (and collaborating centers, if any). The study has been reviewed and approved by the relevant ethics committees before starting.

Procedures (What Happens If You Participate)

If you agree to participate, **you will continue to undergo all the standard tests and procedures for your condition** as planned by your doctors (this typically includes a prostate MRI and possibly a biopsy if the MRI shows suspicious findings). The **only difference** with being in this study is:

- During your scheduled prostate MRI exam, the MRI team will perform an **additional MRI sequence** that lasts about **4–5 minutes**. This special sequence (time-dependent diffusion MRI) will be done **in the same session** as your regular

MRI, immediately after or before the standard MRI sequences. You do not need to come for an extra visit; the MRI appointment will just be extended by a few minutes [~5 minutes] to include this research sequence.

- This additional MRI sequence does not require any extra injections or medications. It involves the MRI scanner taking a few more images. You will likely not notice any difference except a slightly longer time in the scanner. The procedure (lying still in the MRI machine) remains the same as for the standard MRI.
- After the MRI, you will continue with the usual care. If your standard MRI results indicate that a prostate biopsy is needed (as is routine in diagnosing prostate cancer), you will undergo that biopsy as per your doctor's recommendation. **The decision to perform a biopsy or any treatment will be made by your treating physicians based on standard practice, not influenced by the research MRI sequence or AI findings.**
- The research team will collect information from your medical records related to the study, such as the results of your standard MRI, the findings of the new MRI sequence, and the outcome of any prostate biopsy or other relevant tests. The study investigators and the AI software will analyze the images and data to evaluate the new technique's accuracy.

Duration: Your participation in the research will cover the period needed to complete your diagnostic procedures and allow the research team to gather necessary data. In most cases, all study-related activities (the extra MRI sequence and collection of your results) will be completed on the day of your MRI and, if applicable, the day of your biopsy. We may

review your health status or medical records afterward (for example, to record biopsy results or any follow-up information), but **no additional hospital visits or procedures are required** specifically for this study beyond the extra MRI imaging during your scheduled scan.

Risks and Discomforts

Participating in this study **is not expected to involve significant risks** beyond those of your standard medical care. The MRI technology used in this study is non-invasive (it uses magnetic fields, not radiation). The additional MRI sequence is similar to standard MRI sequences. However, we want you to be aware of the following:

- **MRI Scan Discomfort:** During the MRI, you must lie still inside a tunnel-like scanner. The extended scan time (about 5 extra minutes) could cause minor discomfort such as muscle stiffness from lying still or feelings of anxiety/claustrophobia in the confined space. The MRI machine also makes loud knocking noises; you will be provided ear protection as per standard practice. If you feel anxious or uncomfortable at any point, you can communicate with the MRI technician via the built-in intercom.
- **Contrast Dye (if used):** In a standard prostate mpMRI, a contrast dye is often injected into a vein to improve imaging (this is part of routine care, not specifically for this study). The use of contrast can very rarely cause allergic reactions or other side effects. These risks **exist with or without** the research, as contrast is a routine part of the MRI you would undergo. The study's additional MRI sequence does **not** require extra contrast beyond what is normally used.

- **Biopsy Risks (if you have a biopsy):** If your doctors decide you need a prostate biopsy (as part of standard care), that procedure carries its own risks such as bleeding, infection, pain, or urinary difficulties. These risks are **not caused by the research** but are part of the usual medical procedure. Your doctors will explain those risks to you as part of your standard treatment plan, regardless of this study.

At this time, we do not anticipate any other risks from the research procedures themselves. **This intervention is not expected to pose any new or significant risk to you.** In the unlikely event that you experience any unexpected problems or side effects that you think might be related to the study's additional MRI sequence, notify the research team as soon as possible. You can reach Dr. **[Baltasar Ramos B]** at **[+569 9102 9017]** to report any such issues or concerns.

Costs

There is no cost to you for participating in this study. All tests and procedures done specifically for the research will be provided **at no charge.** The special MRI sequence and any materials or software used in the study are funded by the researchers or institution. If you receive any bill, it will only be for the standard medical care you would have received even if you were not in the study. Any procedures or services that are part of this research and not part of your usual care will be paid for by the study. Your participation will not result in any additional expense for you or your insurance.

Benefits

You may not receive any direct benefit from participating in this study. Your medical care for your condition will be the same whether or not you participate (you will still get

the necessary tests and treatments as determined by your doctors). The experimental MRI sequence and AI analysis are being evaluated and are not yet proven to improve diagnosis for individual patients.

However, by participating, you are contributing to medical research that may benefit **future patients**. The information gained from this study could help doctors develop better ways to detect prostate cancer earlier or more accurately. In some cases, the additional MRI sequence might provide extra information about your condition, but **any findings from the research MRI will not be used to guide your immediate treatment** because the technique is still experimental. (If the research MRI were to incidentally reveal a critical finding concerning your health, the researchers will discuss with you and your doctor the appropriate next steps. Otherwise, decisions about your care will rely on standard diagnostic results.)

Alternatives to Participation

Participation in this study is optional. **If you decide not to participate, your medical care will not be affected in any way.** You will still receive all the standard recommended diagnostics and treatments for your condition. In other words, you will undergo the usual prostate MRI without the additional research sequence, and any further procedures (such as biopsy or treatment) will proceed as normally planned by your healthcare providers.

The alternative is simply **not to participate in the research.** This means you would receive the current standard-of-care diagnostic pathway for prostate cancer suspicion, which includes the routine multiparametric MRI and any follow-up that your doctors deem necessary (for example, a biopsy if indicated by the standard MRI results). These standard

procedures have known risks or side effects (for example, the contrast used in MRI or the risks of a prostate biopsy, as mentioned above), but those are **independent of this study** and would occur with standard care. Declining to join the study will not incur any penalty or loss of benefits; you will continue to have access to the same medical care as you otherwise would.

Compensation

You will not be paid for participating in this research study. Participation is entirely voluntary and is not associated with any financial compensation. We greatly appreciate your time and contribution to the research.

If you incur any direct expenses as a result of study participation (for example, travel costs for an extra visit specifically required by the study), please inform the research team. In general, this study is designed to coincide with your regular hospital visits, so no extra travel should be necessary. If any unforeseen study-related expenses arise, the research team will discuss reimbursement with you. Otherwise, there are no costs to you and no payments provided for taking part in this study.

Confidentiality

Your privacy and the confidentiality of your personal information will be strictly protected. **Any information collected about you for this study will be kept confidential to the fullest extent allowed by law.** You will be assigned a **study code number**, and your name or any identifying details will not appear in the research data or reports. The data (MRI images, test results, etc.) will be stored securely and only accessible to the research team and authorized personnel who oversee the study.

When the results of this study are published or presented, **your identity will not be revealed.** Scientific publications or presentations will only include summary data, and no information that could personally identify you will be shared.

Organizations that oversee research, such as ethics committees or regulatory agencies, may inspect the study records to ensure the study is conducted properly. These monitors may access your study information *under confidentiality agreements* to verify data accuracy. All individuals who review your records are required to maintain your confidentiality.

In addition, if you agree, we may retain your MRI images or related samples/data for future research use, but always in anonymized form. (You will have the option, at the end of this form, to consent or not consent to allow your data/samples to be kept for future research after the main study is over.)

Additional Information and New Findings

During the course of the study, **if any new information becomes available that might affect your willingness to continue participating,** you or your treating physician will be informed as soon as possible. For example, this could include new insights about risks, or significant improvements in the experimental technique, or any complications observed, etc. You will then be able to reassess your participation in light of this new information.

You are also free to ask questions at any time. If there is anything you do not understand about the study or if you have questions as the study progresses, you can contact the investigator or study staff (see contact information below). We will do our best to keep you updated on any important developments relating to the study.

Voluntary Participation and Withdrawal

Your participation in this research is entirely voluntary. You have the right to decide not to participate at all, or to start the study and then withdraw at any point, **for any reason.** If you choose not to participate or to withdraw early, there will be **no penalty or loss of benefits** that you are otherwise entitled to. Your decision will **not affect the quality of medical care** you receive at our institution now or in the future. You will not be treated differently by your doctors or the hospital if you decide not to participate or if you withdraw.

If you agree to participate and later change your mind, you can **withdraw your consent** and leave the study at any time. To withdraw, you simply need to inform the study investigator of your decision. You may do this in writing (for example, by signing a "Withdrawal of Consent" form, provided at the end of this document) or by directly communicating with the research team. We also ask that you inform your treating physician if you decide to withdraw, so they are aware you are no longer part of the research (this will have no effect on your regular treatment).

If you withdraw from the study, there will be no negative consequences for you. You will continue to receive normal medical care. **Any data or samples collected from you up to the point of withdrawal will remain part of the study analysis** unless you request that they be destroyed. You will have the option, when withdrawing, to specify whether the research team may still use the data/samples collected before you withdrew (under strict confidentiality) or whether you do not allow any further use of them.

In some circumstances, **the investigator or your doctor might decide to withdraw you from the study** in your best interest. For example, if continuing in the study is deemed harmful to you, if you are unable to complete the MRI procedure, or if you no longer meet

the study requirements, the study team may end your participation. If this happens, it will be explained to you, and it will also not affect your subsequent medical care in any way.

Research-Related Injury or Complications

This study is low-risk and non-interventional, so physical injury from the research is highly unlikely. No experimental drug or invasive procedure is being tested. The main research intervention is an imaging procedure (MRI sequence) similar to those routinely used. **We do not expect any complications directly caused by the additional MRI scanning.**

However, we want to ensure you are protected. In the **unlikely event that you suffer any injury or complications as a direct result of the research procedures** (for example, an unforeseen problem caused by the experimental MRI sequence), you will receive prompt and appropriate medical treatment. The cost of any such treatment will be **covered by the study (or the institution)**, and you will **not** be charged for treatment of research-related injuries.

This coverage applies only to injuries **directly resulting from the study procedures** and not to any complications that are part of your underlying disease or standard medical treatment. (For instance, complications from your prostate cancer or its standard treatment would not be considered "research-related" and would be managed as part of your regular care.) The sponsor/institution has provisions to handle research-related harm; if applicable, an insurance policy is in place to compensate for any research-caused damages. Details of this coverage can be provided to you upon request.

It is important that you promptly report any health issues that you believe might be linked to the study to the investigator (contact information below). By signing this form, you are

not waiving any legal rights or releasing the investigators or institution from liability for negligence.

Participant Rights and Contact Information

As a research participant, you have certain rights, including the right to have your questions answered and the right to withdraw as described above. You will receive a **copy of this informed consent form**, signed by you and the investigator, for your records.

If you have **questions or need more information** about this study at any time, or if you experience any research-related health issues, you may contact the principal investigator:

- **Principal Investigator:** Dr. **[Investigator's Name]** – Department of **[Dept Name]**, HCUCH
  Phone: [Phone Number] (you may call at any time; for urgent issues, this number is answered 24/7 if the study is interventional)
  Email: [Email Address] (if applicable)

You may also contact **[Study Coordinator or Co-Investigator Name]** at [Phone] for general inquiries.

If you have questions about your **rights as a participant in a research study**, or if you have concerns or complaints about the research, you can contact the Research Ethics Committee independent of the research team:

- **Scientific Ethical Committee, Hospital Clínico Universidad de Chile (HCUCH)** – Tel: **(+56 2) 2978 9008**, Email: **comiteetica@hcuch.cl**. Address: Dr. Carlos Lorca Tobar No. 999, 4th Floor (Sector D), Independencia, Santiago, Chile.

(This is the hospital’s ethics committee that oversees research studies. If you have any concerns about the study or your rights as a participant, you may contact them. They are not involved in the conduct of this study and can provide independent advice.)

Note: If other ethical or regulatory committees oversee this research, their contact information will be provided to you as needed.

Consent Statement

Please read the statement below and sign to indicate your agreement:

I have read and understood the information in this consent form (or it has been read to me). All my questions about the study have been answered to my satisfaction. I understand that my participation is voluntary and that I am free to withdraw at any time without affecting my medical care or legal rights. I voluntarily agree to participate in the research study titled "**Non-Invasive Detection of Prostate Cancer with Novel Time-Dependent Diffusion MRI and AI-Enhanced Quantitative Radiological Interpretation (PROSTDAI)**."

By signing this form, I have not waived any of my legal rights. I will receive a copy of this signed consent form for my records.

Participant: *(Please print and sign your name)*

- Name: ___________________________________
- Signature: ______________________________ Date: ___________ Time: ________
- ID Number (RUN or Passport): ___________________

Principal Investigator (or Designee):

- Name: ___________________________________
- Signature: ______________________________ Date: ___________ Time: ________

Institutional Representative

- Name: ___________________________________
- Signature: ______________________________ Date: ___________ Time: ________

Witness (if applicable): *Only required if the participant is illiterate, visually impaired, or otherwise unable to personally read/complete the form.*

- Name of Independent Witness: ___________________________________
- Relationship/Role (e.g., impartial third party or legal representative): ___________________
- Signature of Witness: ___________________________ Date: ___________ Time: ________

Revocation of Consent (Withdrawal Form) – PROSTDAI Study

This section can be used if you decide later to withdraw your consent to participate. You do not need to fill this out now. In case you choose to leave the study, you may complete this form at that time.

I, __________________________________, hereby **voluntarily withdraw** my consent to participate in the **PROSTDAI research study**, and I request to end my participation in the study as of **Date: ____________**. I understand that this decision will not affect my medical care or result in any penalty or loss of benefits.

Regarding the data and/or biological samples that have been collected from me up to this point, **I acknowledge that:** *(Please initial one of the options below)*

- ______ **I ACCEPT** that the research team may **retain and continue to use** my previously collected data and samples **under strict confidentiality** for the purposes of this research (and related future research, if applicable).
- ______ **I DO NOT ACCEPT** the continued use of my data and samples. I request that any of my identifiable data and remaining samples be destroyed or no longer used for research purposes. *(Note: Data or samples that have already been analyzed and included in aggregate results up to this point cannot be removed, but no further data will be collected from me.)*

Participant (withdrawing):

- Name: ________________________________
- Signature: ___________________________ Date: ___________ Time: ________

- ID Number (RUN or Passport): ________________

Principal Investigator (or Designee):

- Name: _______________________________
- Signature: __________________________ Date: ___________ Time: ________

(A signed copy of this revocation form will be provided to the participant. The original will be retained in the study records.)

Witness (if applicable for withdrawal): *(Required if the participant withdrawing is unable to read or sign the form on their own)*

- Name of Witness: ___________________________
- Signature of Witness: ______________________ Date: ___________ Time: ________

Thank you for considering participation in this study. Your contribution is valuable to us. Please do not hesitate to ask any questions or clarify any points before signing. Your understanding and comfort with the research are our top priority.
